# The causal relationship between autoimmune diseases and osteoporosis: a study based on Mendelian randomization

**DOI:** 10.3389/fendo.2023.1196269

**Published:** 2023-08-29

**Authors:** Shaofeng Wu, Zhen Ye, Yi Yan, Xinli Zhan, Liang Ren, Chenxing Zhou, Tianyou Chen, Yuanlin Yao, Jichong Zhu, Siling Wu, Fengzhi Ma, Lu Liu, Binguang Fan, Chong Liu

**Affiliations:** ^1^ Department of Spine and Osteopathy Ward, The First Affiliated Hospital of Guangxi Medical University, Nanning, China; ^2^ Department of Operating Room, Taixing People’s Hospital, Taixing, China; ^3^ Reproductive Medicine Center, The First Afliated Hospital of Guangxi Medical University, Nanning, China

**Keywords:** two-sample Mendelian randomization, osteoporosis, bone mineral density, fall risk, fracture risk

## Abstract

**Objective:**

The relationship between different autoimmune diseases and bone mineral density (BMD) and fractures has been reported in epidemiological studies. This study aimed to explore the causal relationship between autoimmune diseases and BMD, falls, and fractures using Mendelian randomization (MR).

**Methods:**

The instrumental variables were selected from the aggregated statistical data of these diseases from the largest genome-wide association study in Europe. Specifically, 12 common autoimmune diseases were selected as exposure. Outcome variables included BMD, falls, and fractures. Multiple analysis methods were utilized to comprehensively evaluate the causal relationship between autoimmune diseases and BMD, falls, and fractures. Additionally, sensitivity analyses, including Cochran’s Q test, MR-Egger intercept test, and one analysis, were conducted to verify the result’s reliability.

**Results:**

Strong evidence was provided in the results of the negatively association of ulcerative colitis (UC) with forearm BMD. UC also had a negatively association with the total body BMD, while inflammatory bowel disease (IBD) depicted a negatively association with the total body BMD at the age of 45–60 years. Horizontal pleiotropy or heterogeneity was not detected through sensitivity analysis, indicating that the causal estimation was reliable.

**Conclusion:**

This study shows a negative causal relationship between UC and forearm and total body BMD, and between IBD and total body BMD at the age of 45–60 years. These results should be considered in future research and when public health measures and osteoporosis prevention strategies are formulated.

## Introduction

Osteoporosis, a common systemic bone disease, is mainly characterized by reduced bone mineral density (BMD), bone tissue deterioration and structure destruction, and increased risk of fractures (1, 2). Osteoporosis is an increasingly critical public health challenge worldwide. There is a significant increase in the incidence rate with age, and leads to hip fracture, which leads to an increase in mortality (3, 4). However, the pathogenesis and risk factors of osteoporosis are still poorly comprehended; therefore, prevention and treatment remain a huge challenge. Currently, an effective method for diagnosing osteoporosis and evaluating the risk of brittle fractures is through BMD measurement (5). BMD is a feature with highly polygenic influence, as depicted by the Genome-wide association study (GWAS), and some of the genetic determinants may cause low BMD, leading to fractures (6, 7).

Autoimmune diseases have been reported to have a significant impact on bone health, leading to an enhanced osteoporosis and fracture risk; however, the exact mechanism is ambiguous (8). Chronic inflammation associated with autoimmune diseases can damage bone and reduce bone density, making it more prone to fractures (9). Additionally, autoimmunity can lead to an overall decrease in calcium levels through a decrease in calcium absorption and an increase in calcium excretion (10). Although epidemiological studies have shown some association between autoimmune diseases and osteoporosis, whether these associations are causal should be further explored. Unmeasurable confounding factors and reverse causal relationships limit traditional epidemiological research, possibly leading to deviations, thus, hindering the possibility of inferring causal relationships (11). Nevertheless, the determination of whether a causal relationship exists between autoimmune diseases and osteoporosis can point out specific biological pathways and provide information for prevention strategies.

A new method of epidemiological etiology inference is Mendelian randomization (MR), which can effectively offset the bias caused by confounding factors and reverse causality by using genetic variation as an instrumental variable (IV), assist in identifying the causal relationship between exposure and results, and modify the genetic mechanism of human genetics and complex diseases (12, 13). Additionally, GWAS provides a robust and reliable IV for MR research using a genetic variation. Alleles follow the law of independent classification and can reduce the confounding effect of environmental factors, which mimics the design of randomized controlled trials (RCT) (14). In recent years, the causal relationship between multiple complex diseases, including autoimmune diseases, has been assessed widely using MR methods (15, 16).

Thus, we used summary statistics from the largest available genome-wide datasets in this study and applied the MR method to determine the evidence of causal relationships between autoimmune diseases (celiac disease (CeD), rheumatoid arthritis (RA), Crohn’s disease (CD), psoriasis (PsO), primary sclerosing cholangitis (PSC), asthma, inflammatory bowel disease (IBD), primary biliary cirrhosis (PBC), ulcerative colitis (UC), type 1 diabetes (T1D), systemic lupus erythematosus (SLE), ankylosing spondylitis (AS)) and BMD, falls, and fractures. The term “autoimmune” will be used to describe both immune and inflammatory disorders (such as asthma).

## Materials and methods

### Research design

**Figure 1:**
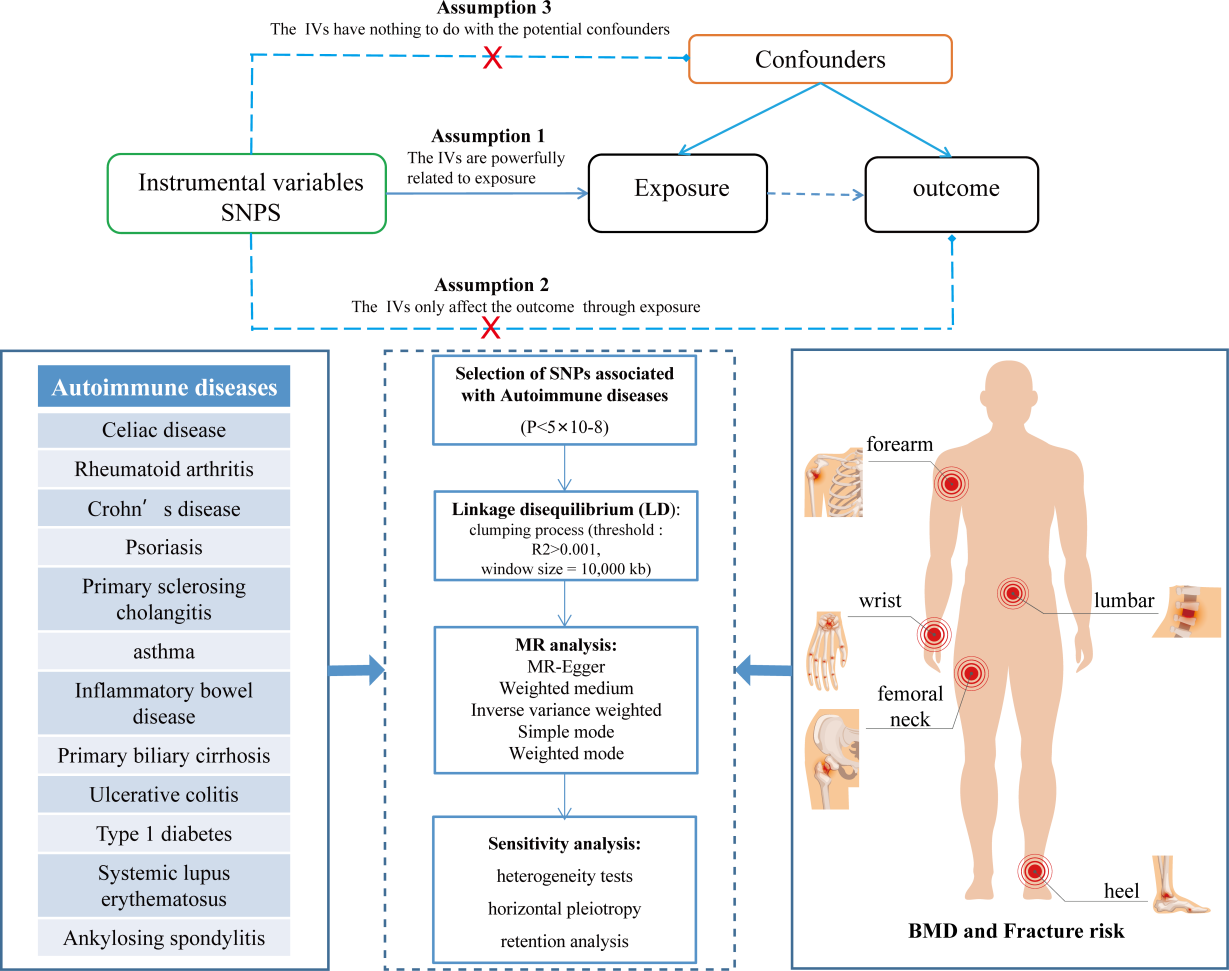
The study design of two-sample MR analysis.

This dual-sample MR study aimed to explore the potential causal relationship between autoimmune diseases and the risk of BMD, falls, and fractures. The single nucleotide polymorphism (SNP) used as a tool must fulfill the following assumptions to obtain convincing conclusions: 1.The relevance assumption, IVs must be strongly associated with exposure; 2. The exclusion restriction assumption, IVs should only affect the outcomes through exposure; 3. The independence assumption, IVs cannot be associated with any possible confounding factors (17). These three key assumptions are illustrated in **Figure 1**. MR analysis strictly follows the Strengthening Epidemiological Observation Research Report (STROBE) guidelines (18). All original studies obtained ethical recognition and informed consent.

### Data resources

Summary-level data from the largest publicly available GWAS for MR analysis of each trait was used for the exposure. The IEU OpenGWAS project provided the summary statistical data of 12 common autoimmune diseases, including CeD, RA, CD, PsO, PSC, asthma, IBD, PBC, UC, T1D, SLE and AS (19). All study participants were of European origin. The diagnostic criteria of the disease are presented in the original publication.

For the outcome, BMD loss in the forearm, femoral neck, lumbar spine, and heel enhances the risk of osteoporosis and fracture more than the other body parts (20). Therefore, the summary statistical data (21) of BMD of the forearm, femoral neck, and lumbar spine were obtained from the GEnetic Factors for OSteoporosis Consortium (GEFOS), including 8143, 32735, and 28498 participants. The GWAS dataset (22) of the UK Biobank provided the summary statistics of heel BMD, including 426,824 participants. The BMD measurement method of the study participants is described in detail in the original publication. Age has been considered a risk factor for osteoporosis (23). Therefore, a large GWAS meta-analysis was used for obtaining the summary statistical data (24) of the whole body BMD of these five age groups, that is, under 15, 15–30, 30–45, 45–60, and over 60 years, including a total of 66,628 participants. Osteoporosis results in fractures of the fragile bones (1). Therefore, the summary statistical data of fractures of various parts such as the arm, ankle, leg, spine, wrist, and other bones were obtained from the MRC IEU OpenGWAS database, including 460,340 and 306,379 participants with fractures of the heel (left and right heels). Additionally, the summary statistical data of falls from the MRC IEU OpenGWAS database was also obtained, including a total of 461,725 participants (25, 26).

### Genetic tool variable selection

After obtaining GWAS summary data of various autoimmune diseases, a series of quality control steps were carried out to ensure that SNPs identified as IV must be closely related to exposure. First, SNPs with genome-wide significance were extracted (P<5×10^-8^). Secondly, to avoid bias, we prune SNPs with r^2^<0.001 threshold with a 10,000 kb window to exclude SNPs in strong linkage disequilibrium (27, 28). Additionally, SNPs corresponding to exposure-related phenotypes were removed through Phenoscanner V2 to eliminate potential pleiotropic effects. Finally, the variance proportion of the exposure is calculated using the R^2^ value of each SNP, and instrument bias is avoided by using *F*-statistics to estimate the instrument strength (29).

### Statistical analysis

In this study, MR-Egger, weighted median (WM), inverse variance weighted (IVW), simple mode, and weighted mode were used to conduct MR analysis to better evaluate the causal relationship between autoimmune diseases and BMD, falls, and fractures. When the level of pleiotropy of IVs does not exist, IVW has the highest statistical power; therefore, it is used as the main analysis method (30, 31). Different types of genetic pleiotropy are considered by other MR methods and are based on potentially different assumptions to check the robustness of the results. For example, WM can still provide unbiased causal estimation, when more than 50% of the information comes from invalid IVs (32). the MR-Egger method can also provide an unbiased estimation of causality when all IVs do not meet the core MR hypothesis (33). The effect of exposure on outcome was expressed using an odds ratio (OR) and 95% confidence interval (95% CI).

### Sensitivity analysis

A series of sensitivity analyses, including heterogeneity tests (IVW and MR-Egger), horizontal pleiotropy (MR-Egger intercept and MR-PRESSO), and retention analysis, were conducted to rule out the possible violation of the MR hypothesis (**Figure 1**). The IV and size of heterogeneity are tested using the *p*-value of Cochran’s Q statistics evaluated by *I^2^* statistics (34). The outliers are detected using MR-PRESSO analysis (35). The level of pleiotropy is identified from the deviation between intercept and zero in MR-Egger analysis (33). Additionally, an analysis is left to evaluate whether the causal effect estimation may be biased or interfered with by a single SNP (30). The results are visualized using funnel and forest plots. All MR analyses were performed using the “TwoSampleMR” package in R version 4.2.1. *P*<0.05 is considered statistically significant.

## Results

### The causal relationship between autoimmune diseases and bone mineral density

**Figure 2:**
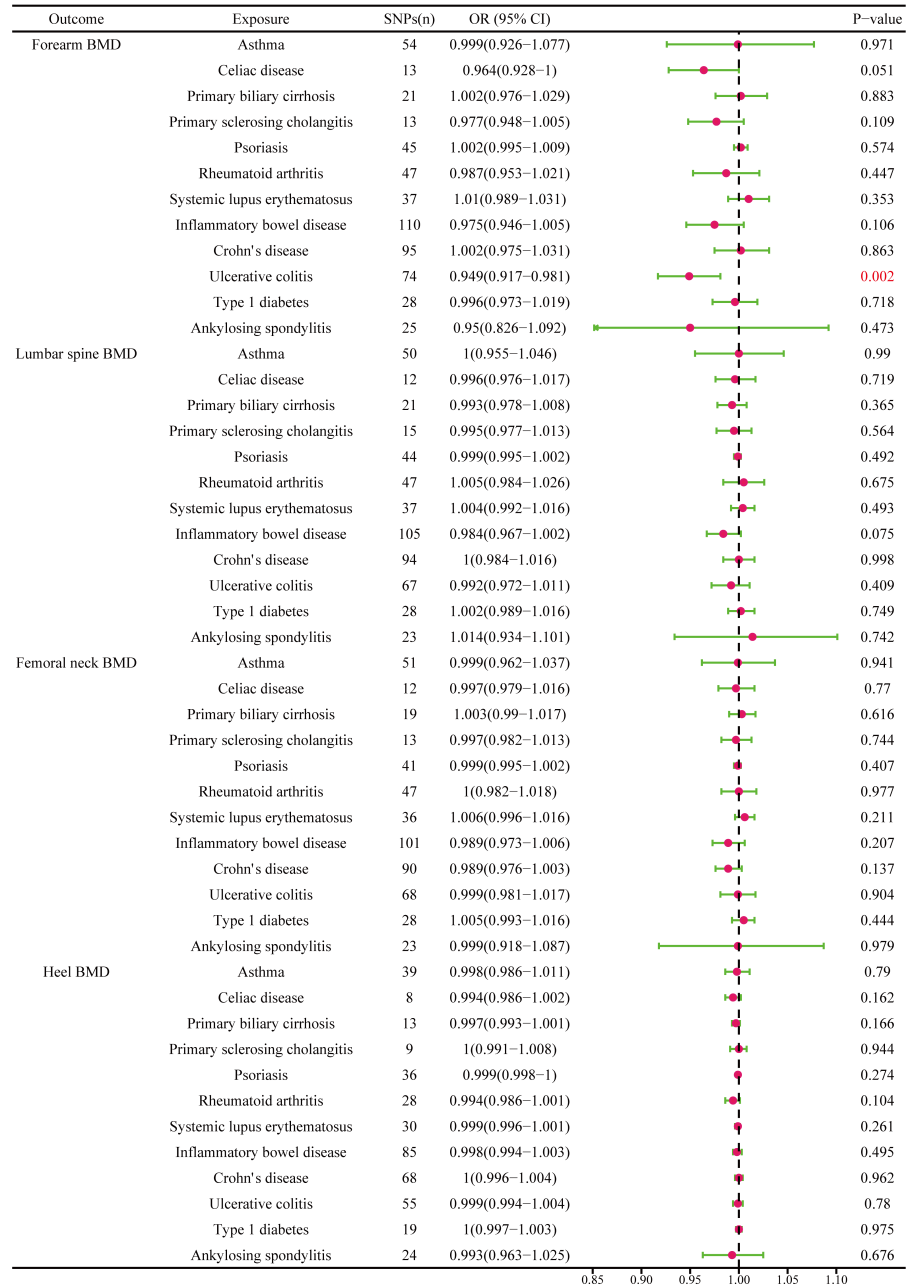
The causal effect of autoimmune diseases on different parts of the body BMD. IVW, inverse variance weighted; CI, confidence interval; BMD, bone mineral density; SNP, single nucleotide polymorphism.

**Figure 3:**
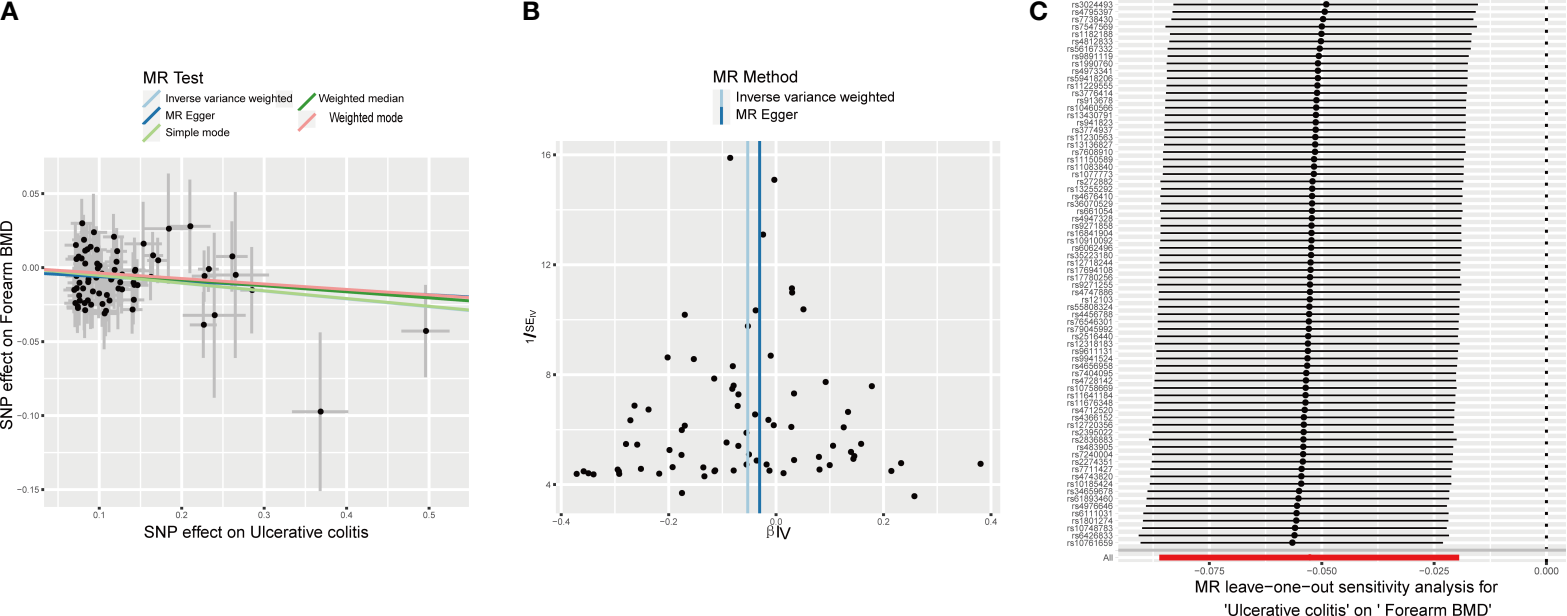
Effects of UC on forearm BMD. **(A)** Scatter plot. The slopes of each line represent the causal association for each method. **(B)** Funnel plot. **(C)** Leave-one-out analysis.

The different body regions have different causal effects of autoimmune diseases on BMD. Based on the IVW results, UC was negatively associated with BMD of the forearm (OR = 0.949, 95% CI = 0.917–0.981, P = 0.002) **(** **Figure 2** **)**. In the analysis of the heterogeneity test, neither IVW nor Cochran’s Q test of the MR-Egger method found evidence of heterogeneity. Directional pleiotropy was not demonstrated using the MR-Egger intercept test results. Additionally, the remaining analysis also proved that the robustness of MR affected estimation **(** **Figure 3** **) (** **Table 1** **)**. However, the IVW method did not demonstrate any statistically significant relationship between other autoimmune diseases and BMD in different body regions, and the robustness of MR affecting estimation was proved by the sensitivity analysis (**Supplementary Material**).

**Table 1:** Heterogeneity and pleiotropy test.

Exposure	Outcome	Heterogeneity test						Pleiotropy test		
		MR Egger			Inverse variance weighted			MR Egger		
		Q	Q_df	*p*-value	Q	Q_df	*p*-value	Intercept	SE	*p*-value
Ulcerative colitis	Forearm BMD	54.46708708	72	0.939	54.81975559	73	0.945	-0.003004044	0.005058513	0.554
Ulcerative colitis	Total body BMD	62.10969646	71	0.765	62.11729292	72	0.791	0.000165392	0.001897618	0.931
Inflammatory bowel disease	Total body BMD of age 45-60	81.18113323	101	0.926	81.19422407	102	0.936	0.000316064	0.002762434	0.909
Rheumatoid arthritis	Fall risk	42.10563381	42	0.466	42.84657138	43	0.478	-0.000301325	0.000350501	0.395
Type 1 diabetes	Fall risk	19.48880477	28	0.882	19.49582075	29	0.908	-3.79921E-05	0.000453575	0.934
Primary sclerosing cholangitis	Wrist fracture risk	8.720075514	11	0.648	8.723253192	12	0.726	-1.00249E-05	0.000177838	0.956
Inflammatory bowel disease	Left heel fracture risk	103.0394544	115	0.78	103.2340246	116	0.796	3.73355E-05	8.46416E-05	0.66
Crohn’s disease	Left heel fracture risk	96.69719326	102	0.63	97.43887134	103	0.636	9.14191E-05	0.000106152	0.391
Ulcerative colitis	Left heel fracture risk	64.18085106	77	0.851	64.36435388	78	0.866	4.60851E-05	0.000107582	0.67

MR, Mendelian randomization; Q, heterogeneity statistic Q; df, degree of freedom; SE, standard error.

**Figure 4:**
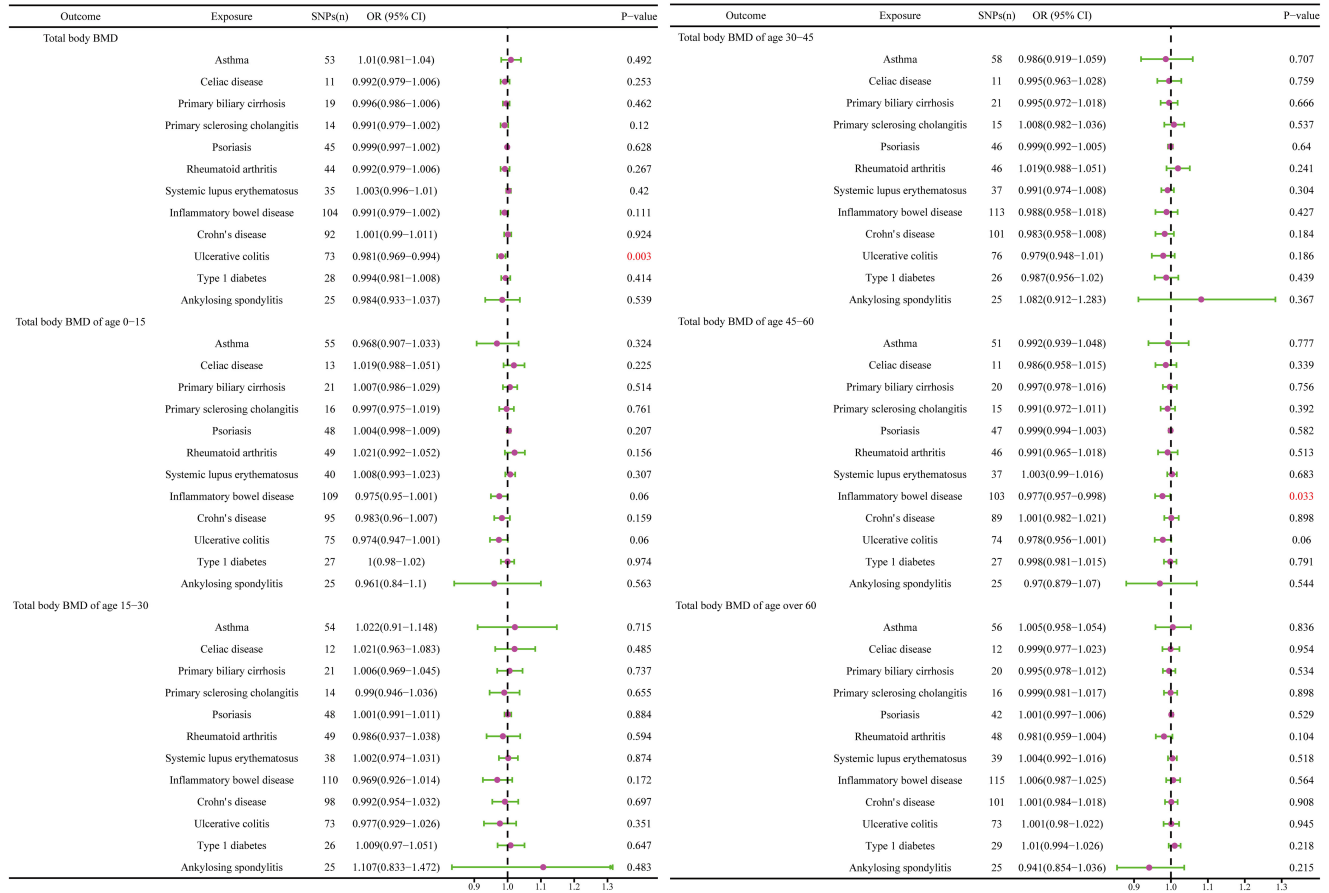
The causal effect of autoimmune diseases on total body BMD and that at different ages. IVW, inverse variance weighted; CI, confidence interval; BMD, bone mineral density; SNP, single nucleotide polymorphism.

**Figure 5:**
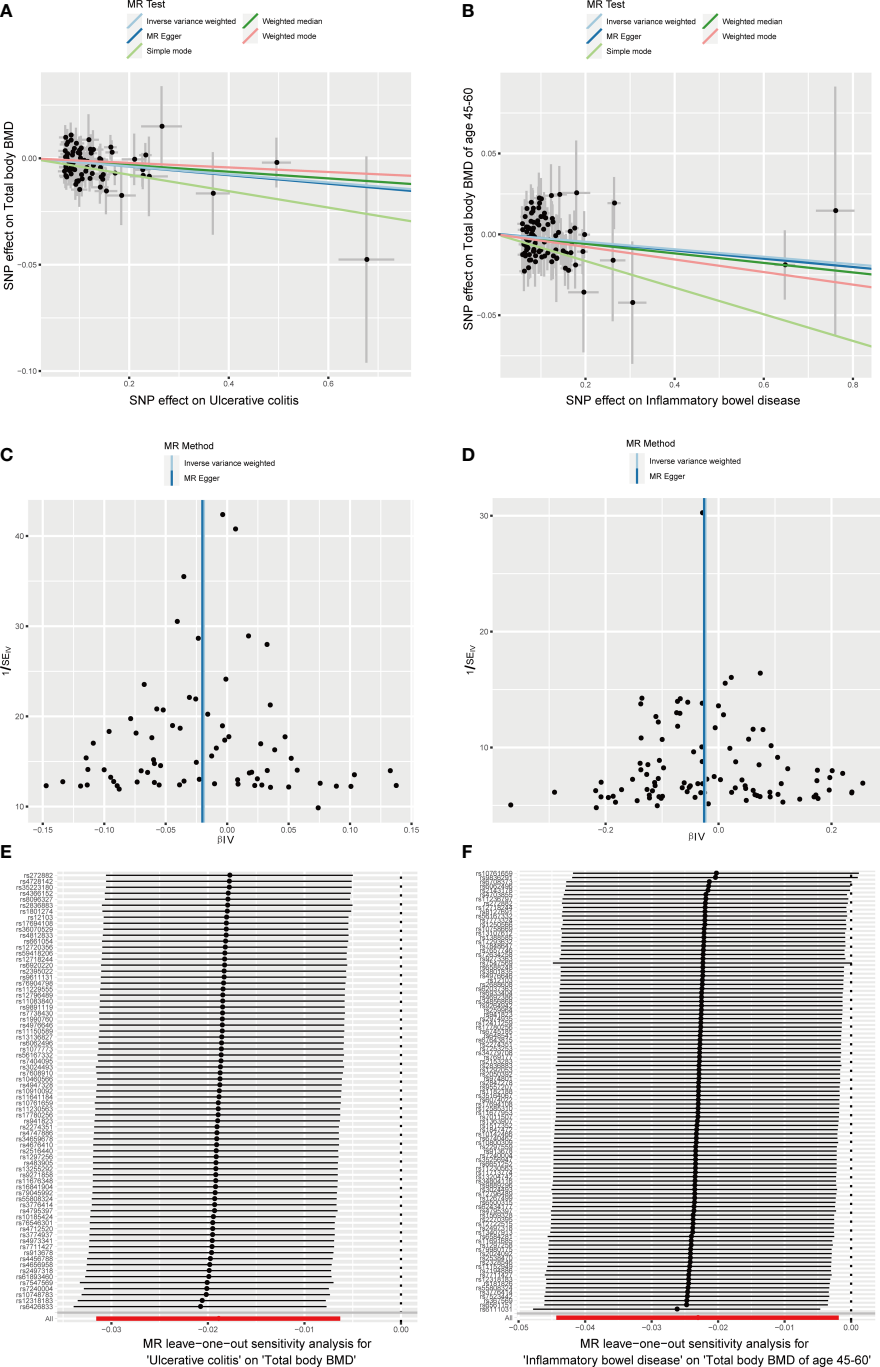
Effects of UC and IBD on total body BMD and that at different ages. **(A, B)** Scatter plot. The slopes of each line represent the causal association for each method. **(C, D)** Funnel plot. **(E, F)** Leave-one-out analysis.

The causal effect of autoimmune diseases on total body BMD and that at different ages. Based on the IVW results, UC was negatively associated with the total body BMD (OR = 0.981, 95% CI = 0.969–0.994, P = 0.003), while IBD was negatively associated with the total body BMD at the age of 45–60 years (OR = 0.977, 95% CI = 0.957–0.998, P = 0.033) **(** **Figure 4** **)**. No evidence of heterogeneity was found using Cochran’s Q test of IVW and MR-Egger methods. Directional pleiotropy was not demonstrated by the MR-Egger intercept test results. Additionally, the robustness of MR effect estimation was also proved by the remaining analysis (**Figure 5**) (**Table 1**). However, no significant relationship existed between other autoimmune diseases and systemic BMD and that of different age groups observed in the IVW method. Sensitivity analysis also proved the robustness of MR effect estimation (**Supplementary Material**).

### The causal relationship between autoimmune diseases and fall risk

**Figure 6:**
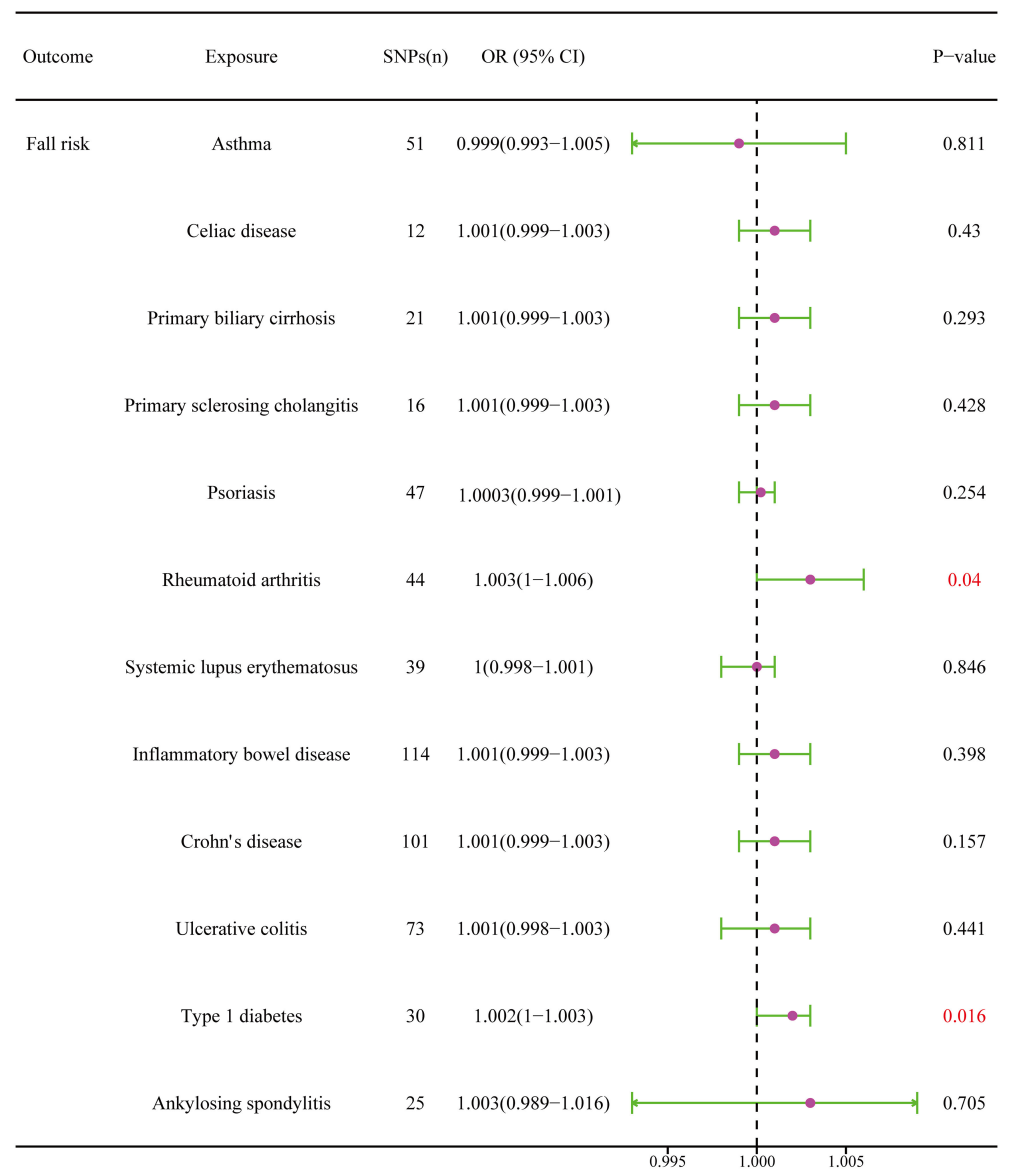
The causal effect of autoimmune diseases on fall risk. IVW, inverse variance weighted; CI, confidence interval; BMD, bone mineral density; SNP, single nucleotide polymorphism.

**Figure 7:**
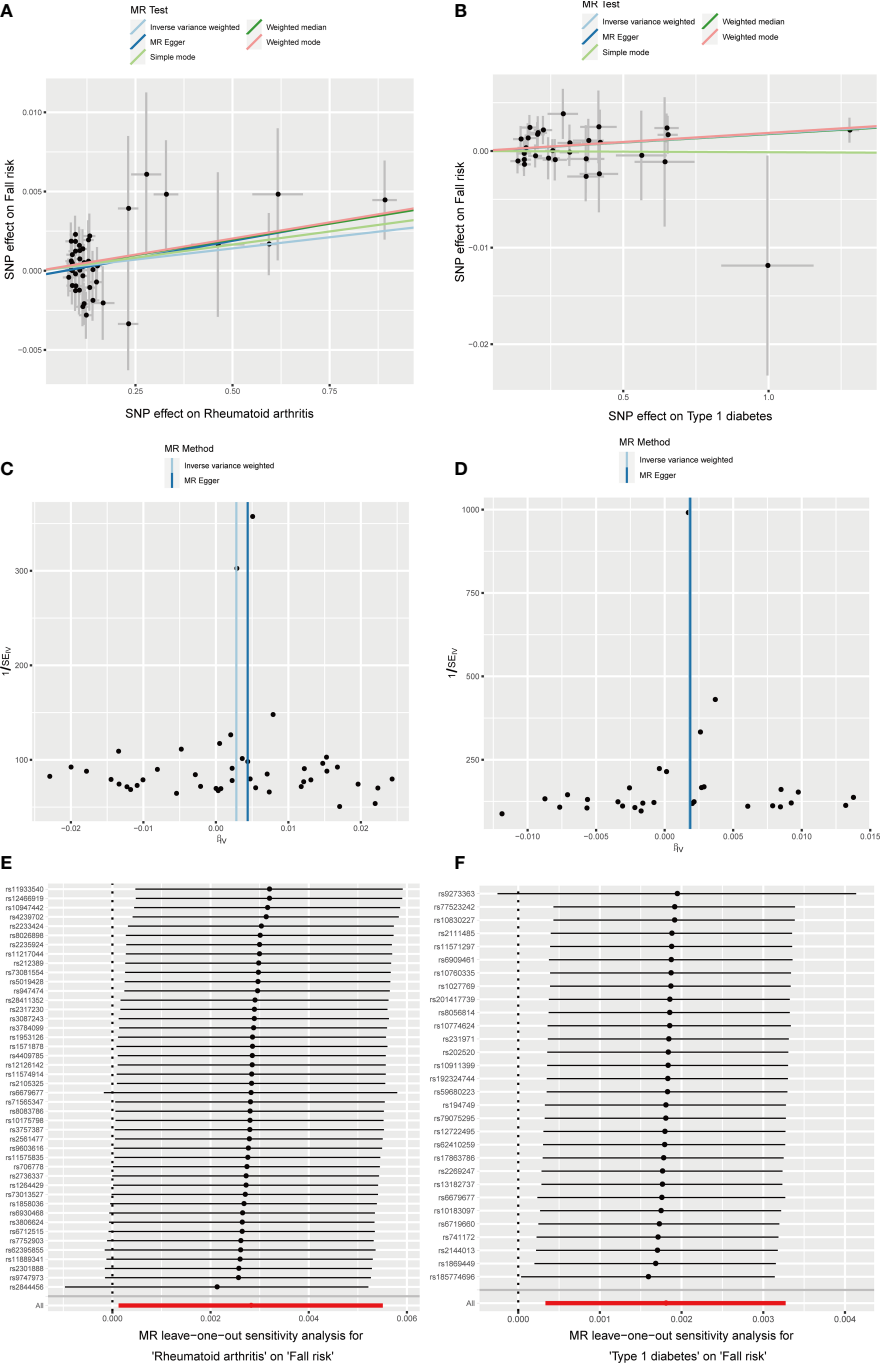
Effects of RA and T1D on fall risk. **(A, B)** Scatter plot. The slopes of each line represent the causal association for each method. **(C, D)** Funnel plot. **(E, F)** Leave-one-out analysis.

RA was positively associated with the falls (OR = 1.003, 95% CI = 1.000–1.005, P = 0.04), which was supported by the weighted median analysis (OR = 1.003, 95% CI = 1.000–1.007, P = 0.049). Additionally, T1D was weakly positively associated with falls (OR = 1.002, 95% CI = 1.000–1.003, P = 0.016) (**Figure 6**). No evidence of heterogeneity was detected using Cochran’s Q test of IVW and MR-Egger methods. Directional pleiotropy was not demonstrated using MR-Egger intercept test results. Additionally, the remaining analysis also proved the robustness of MR effect estimation (**Figure 7**) (**Table 1**). However, no significant relationship existed between other autoimmune diseases and systemic BMD and that of different age groups observed in the IVW method. Sensitivity analysis also proved the robustness of MR effect estimation (**Supplementary Material**).

### The causal relationship between autoimmune diseases and fracture risk

**Figure 8:**
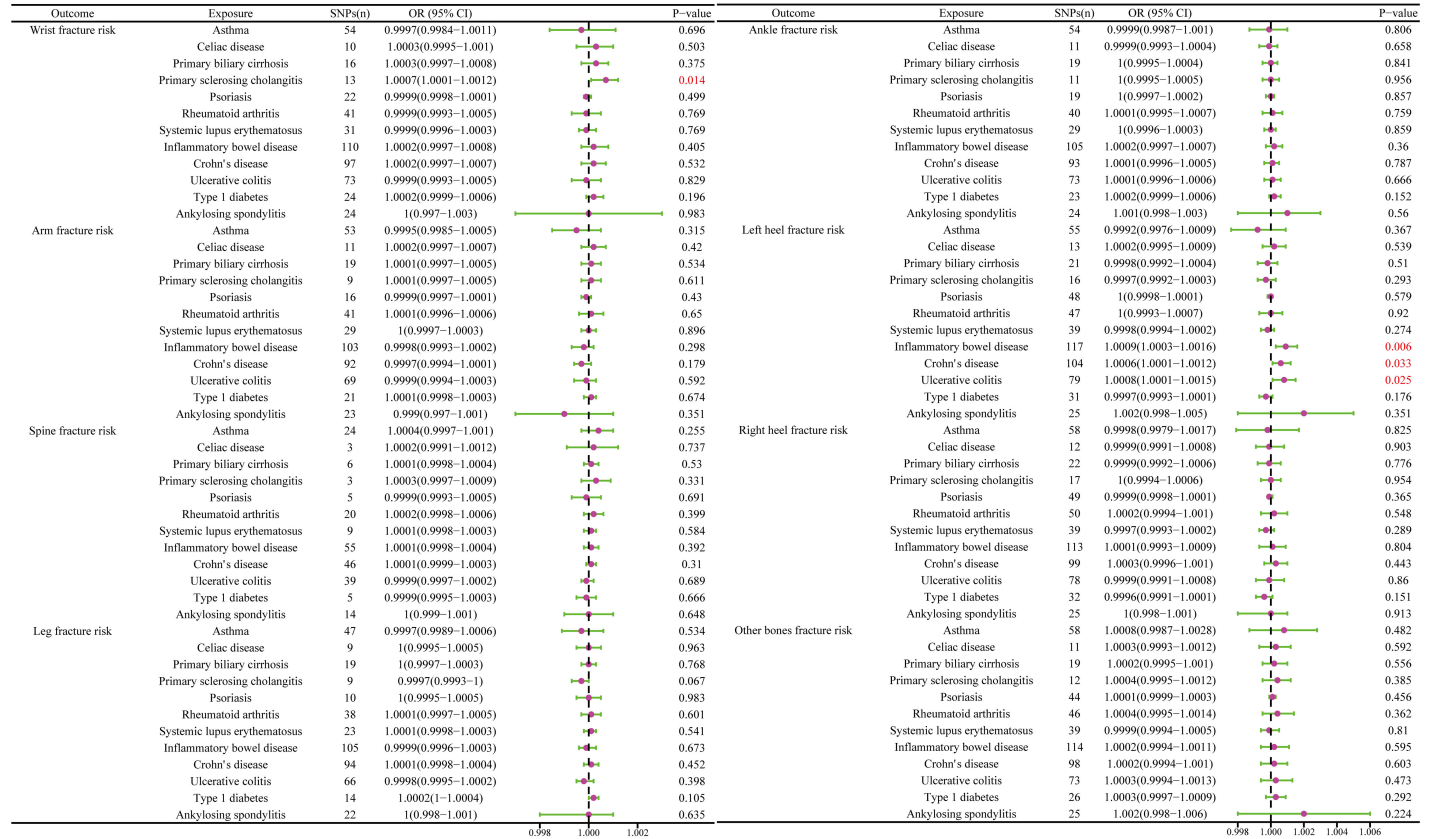
The causal effect of autoimmune diseases on fracture risk. IVW, inverse variance weighted; CI, confidence interval; BMD, bone mineral density; SNP, single nucleotide polymorphism.

**Figure 9:**
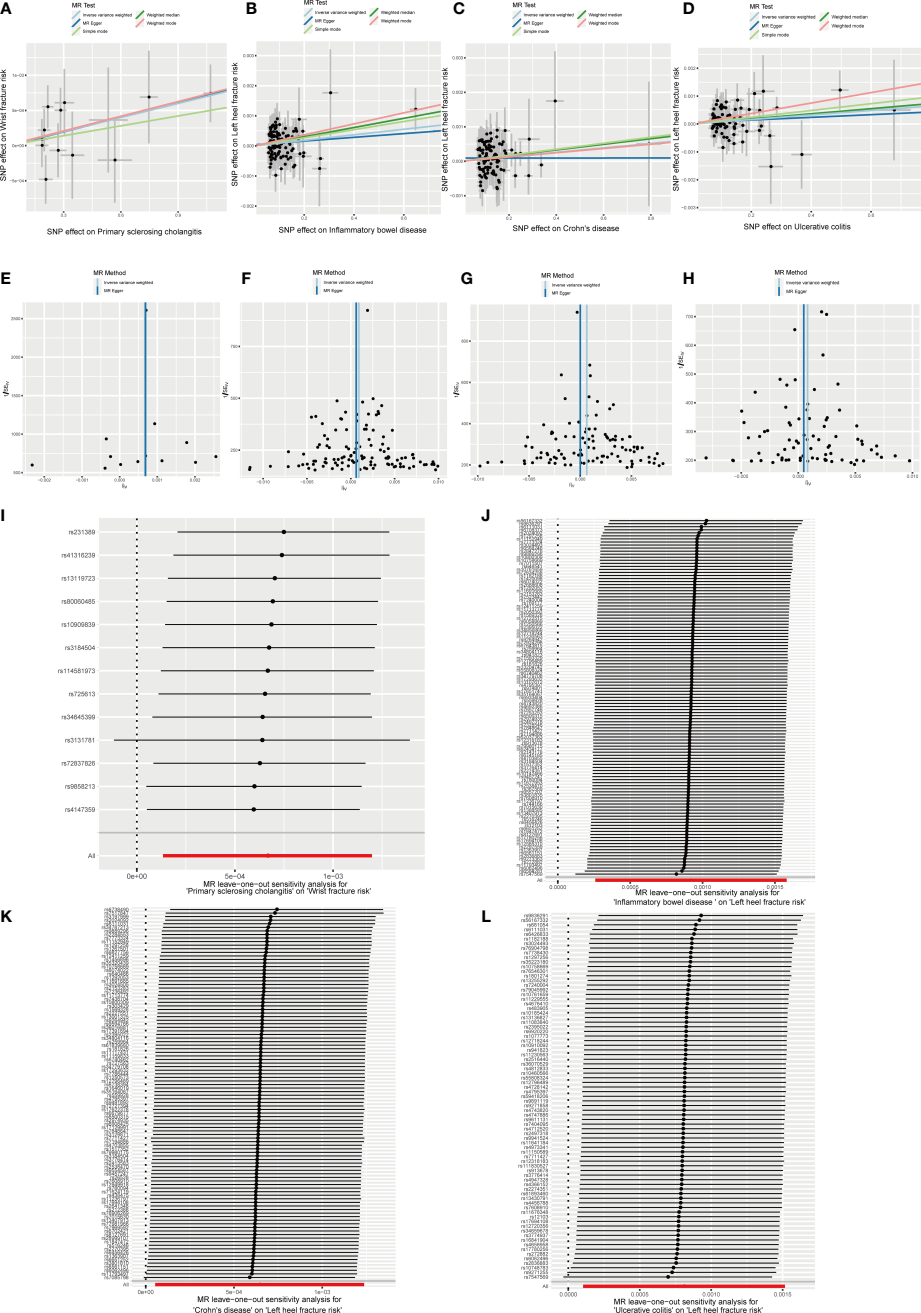
Effects of PSC, IBD, CD and UC on fracture risk. **(A–D)** Scatter plot. The slopes of each line represent the causal association for each method. **(E–H)** Funnel plot. **(I–L)** Leave-one-out analysis.

The MR results between autoimmune disease and fracture risks are shown in the table. Based on the IVW results, PSC was weakly positively associated with the risk of wrist fractures (OR = 1.0007, 95% CI = 1.0001–1.0012, P = 0.014), and several immune diseases were weakly positively associated with the increased risk of left heel fractures, including IBD (OR = 1.0009, 95% CI = 1.0003–1.0016, P = 0.006); CD (OR = 1.0006, 95% CI = 1.0001–1.0012, P = 0.033); and UC (OR = 1.0008, 95% CI = 1.0001–1.0015, P = 0.025) (**Figure 8**). No evidence of heterogeneity was found using Cochran’s Q test of IVW and MR-Egger methods. MR-Egger intercept test results did not show directional pleiotropy. Additionally, the remaining analysis also proved the robustness of MR effect estimation (**Figure 9**) (**Table 1**).

## Discussion

This study aimed to explore the causal relationship between autoimmune diseases and BMD, falls, and fractures. The causal relationship between different immune diseases and BMD in different body regions, different age levels, and falls and fractures in different body parts was assessed using GWAS data, and the two-sample Mendelian randomization analysis was conducted. The study results demonstrated a negative causal relationship between UC and forearm and body BMD, and that between IBD and body BMD at the age of 45–60 years. A weakly positive causal relationship existed between RA and T1D and falls and between PSC and the risk of wrist fractures. There was a positive causal relationship with the risk of left heel fractures. However, there was no evidence to support the causal relationship between other immune diseases and outcomes.

Osteoporosis is mainly characterized by reduced BMD and increased fracture risk. Research shows that many autoimmune rheumatic diseases have been proven to be complicated by systemic and local bone loss (36–38). However, this pathogenic process has multifactorial physiology and pathology, including treatment, fixation, and reduction of physical activity caused by musculoskeletal symptoms (39). BMD reduction in autoimmune diseases is determined by high disease activity, long disease duration, and joint injury (40). Some patients may develop focal or systemic bone loss before the diagnosis of autoimmune diseases. In RA patients without disease-modifying antirheumatic drugs or corticosteroids, BMD is mainly related to demographic factors such as age and gender (40, 41). Thus, these findings suggest that other factors, such as antirheumatic treatment, may partly mediate the reduction of BMD in some patients with autoimmune diseases (42–44). Therefore, whether autoimmune diseases have a direct impact on osteoporosis is still ambiguous.

IBD encompassing both UC and CD, is a chronic, recurrent, inflammatory disorder of the gastrointestinal tract (45). In recent years, the association between IBD and BMD has been widely explored. A prospective study showed BMD reduction in both CD and UC patients (46). Another study found that BMD values of young male patients with IBD not using corticosteroids were lower than those of healthy controls (47). Conversely, Bernstein et al. demonstrated that the decrease in BMD in patients with IBD was associated with the use of corticosteroids but not with the disease itself (48). The main determinants of BMD in patients with CD are sex, age, and body weight (49). This may be the key factor contributing to the discrepancy between the conclusions owing to the presence of many confounders in traditional observational studies. MR analysis based on the random distribution of genetic variation can effectively overcome the bias caused by hybrid and reverse causal problems and provide more reliable results compared with observational research (12). Recently, Wu et al. also used MR to analyze the causal relationship between IBD and BMD. These results support the causal effect of UC on bone BMD reduction in the whole body and forearm BMD. The CD has a clear causal relationship with the reduction of bone BMD in the femoral neck (50). However, this study was limited owing to its small sample size, and only the causal relationship between IBD and BMD in different body regions was explored, without exploring the causal relationship between IBD and BMD at different age levels and the risk of falling and fractures. Therefore, a larger sample size was used, and a more comprehensive assessment was conducted in this study, which not only proved a negative causal relationship between UC and forearms BMD and body BMD but also found a negative causal relationship between IBD and body BMD at the age of 45–60 years. Osteoporosis is an important risk factor for accidents, disability, and death (51). Therefore, this finding is of great significance to public health. Fractures reduce quality of life and independence and increase the risk of mortality. The result is a significant economic and social burden, which will increase even more with an aging population (52). With approximately 18 million wrist and hand fractures worldwide, wrist fractures are the most common type, constituting a huge medical burden (53). Heel fractures account for 1-2% of all fractures, whereas talar fractures account for approximately 60% and have a higher incidence and disability rate, making it a significant public health concern (54–56). Research has found that risk factors for heel fractures include osteoporosis, diabetes, and others (55, 57). In this study, we found a positive causal relationship between PSC and the risk of wrist fractures. Additionally, a positive causal relationship was found between IBD, including UC and CD, and the risk of left heel fractures. However, we noticed that the OR values of these results are very close to 1, and the confidence interval is also very close to 1, indicating that these associations are very weak and may not have clinical significance. Therefore, more research is needed to verify these associations.

Prevention of falls is a major public health challenge with significant associations with immune-mediated diseases. Studies have found that the fall rate among patients with multiple sclerosis is always >50%, leading to a 2–3 fold higher rate of injuries and fractures compared to age-matched controls (58). Kensuke et al.’s study also found that the risk of falls was increased in patients with RA, with reported incidence rates of 30–60% (59, 60) and a higher risk of falls-related fractures than the general population (61). Therefore, the causal relationship between the risk of falls in patients with immune-mediated diseases must be explored to prevent the consequences of falls and treating osteoporosis. Our study found a positive causal relationship between falls and RA and T1D among many immune-mediated diseases, indicating that early interventions are essential to reduce the risk of injury and costly falls.

However, this study has several limitations. First, in the two-sample MR analysis, no overlap of participants should exist between the exposure and outcome samples. However, in this study, we were unable to estimate the degree of overlap. For larger consortia summary statistics with sample overlap to the second dataset used, the direction and extent of bias are unknown, but in very large alliances, the bias of weak tools may not be significant, but in medium-sized alliances, potential biases and exaggerated Type 1 error rates should be investigated (62). Therefore, by calculation, for larger consortia summary statistics with sample overlap to the second dataset used, the bias is 0.001 and the Type 1 error rate is 0.05. In addition, we conducted sensitivity analyses to assess the robustness of our results to different assumptions about the strength of the instruments and the potential for sample overlap (33). Secondly, using summary data from large GWAS datasets cannot analyze stratified risk factors related to disease duration, severity, treatment, and complications. Additionally, most of the participants included in this MR analysis were Europeans; therefore, our conclusions should be validated in other populations. Finally, although measures were taken to exclude outlier variants, the effect of pleiotropic effects on the results could not be excluded; thus, larger sample MR studies or RCTs are needed to obtain more accurate results, and confounding factors may still act through the second hypothesis of MR.

## Conclusion

In conclusion, a negative causal relationship exists between UC and forearm and body BMD, and between IBD and body BMD at the age of 45–60 years. However, no evidence exists to support the causal relationship between other immune diseases and outcomes. These results should be considered in future research and when formulating public health measures and osteoporosis prevention strategies.

## Data availability statement

The datasets presented in this study can be found in online repositories. The names of the repository/repositories and accession number(s) can be found in the article/**Supplementary Material**.

## Ethics statement

All original studies obtained ethical recognition and informed consent. Written informed consent to participate in this study was provided by the participants’ legal guardian/next of kin. Written informed consent was obtained from the individual(s) for the publication of any potentially identifiable images or data included in this article. Ethical review and approval was not required for the study on human participants in accordance with the local legislation and institutional requirements. Written informed consent for participation was not required for this study in accordance with the national legislation and the institutional requirements.

## Author contributions

SFW, CL, and XZ designed the study. LC and JZ analyzed the data. YYan, ZY, CZ, and BF processed the digital visualization. SFW wrote and revised the manuscript. CL, LR and XZ revised the manuscript. All co-authors participated in the laboratory operation. All authors contributed to the article and approved the submitted version.
